# N^6^-Methyladenosine Modification-Related Genes Express Differentially in Sterile Male Cattle-Yaks

**DOI:** 10.3390/life14091155

**Published:** 2024-09-12

**Authors:** Yuxin Liu, Lili Chen, Hui Jiang, Hongzhuang Wang, Yujiao Zhang, Zhengrong Yuan, Yi Ma

**Affiliations:** 1 Institute of Animal Science and Veterinary, Tianjin Academy of Agricultural Sciences, Tianjin 300381, China; yuxinliu22@163.com (Y.L.); chenlili0609@163.com (L.C.); 2 Institute of Animal Sciences, Chinese Academy of Agricultural Sciences, Beijing 100193, China; 3 Tianjin Key Laboratory of Animal Molecular Breeding and Biotechnology, Tianjin 300381, China; 4 Tianjin Engineering Research Center of Animal Healthy Farming, Tianjin 300381, China; 5 Institute of Animal Husbandry and Veterinary Medicine, Xizang Academy of Agricultural and Animal Husbandry Sciences, Lhasa 850002, China; jianghui@taaas.org (H.J.); wanghongzhuang66@163.com (H.W.); 6 College of Biological Sciences and Technology, Beijing Forestry University, Beijing 100083, China; zyujiao@bjfu.edu.cn

**Keywords:** yak, cattle-yak, male sterility, m^6^A modification, m^6^A-related genes

## Abstract

N^6^-methyladenosine (m^6^A), an RNA post-transcriptional modification, plays a crucial role in spermatogenesis. Cattle-yaks are interspecific hybrid offsprings of yak and cattle, and male cattle-yaks are sterile. This study aims to investigate the role of m^6^A modification in male cattle-yak infertility. Herein, testicular tissues were analyzed via histological observations, immunohistochemical assays, reverse-transcription quantitative polymerase chain reaction, Western blotting, and immunofluorescence assays. The results revealed that male cattle-yaks presented smaller testes (5.933 ± 0.4885 cm vs. 7.150 ± 0.3937 cm), with only single cell layers in seminiferous tubules, and weakened signals of m^6^A regulators such as METTL14 (methyltransferase-like 14), ALKBH5 (alpha-ketoglutarate-dependent hydroxylase homolog 5), FTO (fat mass and obesity-associated protein), and YTHDF2 (YTH N^6^-methyladenosine RNA binding protein F2), both at the RNA and protein levels, compared with those of yaks. Altogether, these findings suggest that m^6^A modification may play a crucial role in male cattle-yak sterility, providing a basis for future studies.

## 1. Introduction

Domestic yaks (*Bos grunniens*) of the class Mammalia, order Artiodactyla, and family Bovidae primarily inhabit high-altitude regions such as Qinghai Province, Gansu Province, and the Tibet Autonomous Region. They are important domestic animals in pastoral areas of the Qinghai–Tibet Plateau and can adapt to severe conditions including hyper-cold and hypoxia. They are economic animals and are used for their meat, milk, fur, and labor by local herdsmen. Nevertheless, the quality and quantity of their meat and milk are lower than those of the cattle (*Bos taurus*). Notably, by crossing yaks with cattle, their meat and milk production performance has been considerably improved.

Cattle-yak hybrids (*B. taurus*, ♂ × *B. grunniens*, ♀) are vital to the livestock industry in high-altitude regions due to their superior adaptability and productivity [1]. Nevertheless, a significant challenge in breeding these hybrids is the prevalent issue of male sterility, which limits their reproductive efficiency. Male sterility in cattle-yaks poses a major reproductive barrier, hindering the propagation of this valuable hybrid [2]. Despite its importance, the underlying molecular mechanisms of this sterility remain poorly understood.

Incompatibilities in interspecific hybrids, such as sterility and lethality, are widely observed causes of reproductive isolation and thus contribute to speciation [3]. Hybrid male sterility (HMS) has been a focus in studies of speciation because sterility imposes a barrier to free gene flow between organisms, thus effectively isolating them as distinct species [4]. To date, research on HMS has focused on crosses between horses × asses (*Equus caballus*, ♀ × *Equus asinus*, ♂), lions × tigers (*Panthera leo*, ♀ × *Panthera tigris*, ♂), and cattle × yaks (*B. taurus*, ♂ × *B. grunniens*, ♀). These hybrids’ male offspring are always sterile [5]. Wodsedalek et al. [6] posited that male mule sterility following the hybridization of asses and horses may be attributable to differences in the numbers of chromosomes in these parental species, contributing to meiotic block and impaired spermatogenesis. HMS is a well-documented phenomenon in various species, often linked to genetic and epigenetic factors.

Up to now, there have been many studies on the mechanism of male sterility in cattle-yaks. Despite being similar in appearance, the testes of cattle-yaks are smaller and lighter than those of yaks. Microscopic observations have revealed the absence of mature sperm in seminiferous tubules. Notably, insufficient follicle-stimulating hormone (FSH) and luteinizing hormone (LH) secretion has been considered a contributing factor to sterility [7]. The formation, development, and function of the testes are regulated by the hypothalamic–pituitary–gonadal axis, and the balance of hormone levels is crucial for the normal development of reproductive organs. Nevertheless, reportedly, FSH and LH levels in male cattle-yaks are consistent with those of their parents [8,9]. Moreover, continuous intravenous administration of FSH and LH in well-fed hybrid calves showed increased libido, but no sperm production was observed in seminiferous tubules [10]. Studies at the chromosome level revealed that yaks, cattle, and their breeding generations exhibited the same number of chromosomes as both parental species (2*n* = 60) [11]. Nevertheless, the chromosomes of cattle-yaks are different in the structure of parental species, thus affecting and destroying the normal association, exchange, and separation of chromosomes. With the backcross, the structure of the synaptonemal complex gradually converged with that of the parents [12]. Heterologous hybridization-induced abnormalities in the structure of the synaptonemal complex have been observed. The lack of primary spermatocytes (Pri-SPCs) caused by meiosis abnormality is considered the main cause of sterility [13,14]. With advancements in molecular biology in various fields, genetic research on cattle-yak male sterility is also progressing, including multi-omics analysis and epigenetic modification regulation.

Recent studies have suggested that m^6^A, a prevalent RNA modification, plays a crucial role in regulating gene expression during spermatogenesis [15,16,17,18]. Additionally, a decline in the overall m^6^A methylation level in testicular tissue in cattle-yaks [19], along with changes in the transcriptional level of m^6^A-related genes, has been observed compared with that of the domestic yaks and cattle [20]. Therefore, exploring the mechanism underlying m^6^A modification in male sterility in cattle-yaks is important. While the role of m^6^A in gene regulation is increasingly recognized, its specific contribution to the sterility of male cattle-yaks has yet to be elucidated. This gap in understanding necessitates further investigation into m^6^A-related genes in these hybrids.

The m^6^A RNA modification is one of the most common epigenetic modifications in eukaryotes. It involves the following three types of proteins: methyltransferases, which catalyze methyl group transfer; demethyltransferases, which eliminate added methyl groups; and recognition proteins, which specifically identify m^6^A sites in transcripts [21]. Methyltransferase-like 14 (METTL14), with a molecular weight of 52 kDa, does not independently bind to S-adenosyl methionine (SAM) and thus lacks intrinsic catalytic activity [22]. It pairs with methyltransferase-like 3 (METTL3) to form heterodimer complexes in a 1:1 ratio, offering structural support that stabilizes METTL3 and assists in the recognition and binding of RNA substrates, with both being co-localized in the nuclear speckle region [23]. Demethylases play a pivotal role in the reversible regulation of m^6^A methylation. The two prominent demethylases, fat mass and obesity-associated protein (FTO) and alpha-ketoglutarate-dependent hydroxylase homolog 5 homolog 5 (ALKBH5), are part of the Fe(II) and α-ketoglutarate-dependent ALKB family of dioxygenase proteins. FTO was initially linked to obesity. Further research in 2011 demonstrated that FTO is capable of mediating m^6^A demethylation [24]. ALKBH5, identified in 2013 as a homolog of FTO in mammals, is the second m^6^A demethylase to be discovered. The myriad biological functions associated with m^6^A modifications are mediated by ‘Readers’, a class of proteins that recognize and dictate the destiny of RNA molecules. The first YTH domain-containing proteins identified in mammals are categorized into five types: YTHDF1, YTHDF2, YTHDF3, YTHDC1, and YTHDC2. YTH N^6^-methyladenosine RNA binding protein F2 (YTHDF2), being the first m^6^A ‘Reader’ to be identified, recruits the intracellular mRNA deadenylation complex, known as the carbon catabolite repression 4 (CCR4)-negative on TATA-less (NOT) (CCR4-NOT) deadenylase complex, to collaboratively facilitate the degradation of targeted RNA molecules [25]. Altogether, these proteins affect various RNA metabolic processes, including RNA stability, translation, and alternative splicing events [26].

This study aims to investigate the differential expression of m^6^A-related genes in male yaks and cattle-yaks, hypothesizing that m^6^A modification is critical in mediating hybrid sterility. Understanding the role of m^6^A modifications in cattle-yak sterility could not only provide insights into the mechanisms of hybrid sterility but also inform breeding strategies to overcome reproductive barriers, enhancing the sustainability of these hybrids.

To address this hypothesis, we employed a combination of histological analysis and molecular biological assays to assess the expression patterns of m^6^A-related genes in testicular tissues from male yaks and sterile cattle-yaks.

## 2. Materials and Methods

### 2.1. Ethical Statement

All animal-related procedures were consistent with guidelines established by the China Council on Animal Care and the Ministry of Agriculture of the People’s Republic of China. The Animal Care and Use Committee of the Tianjin Institute of Animal Husbandry and Veterinary Medicine approved all animal handling procedures for this study (2023003).

### 2.2. Animals

Herein, both testes were collected from each of three 3-year-old male yaks (*n* = 3) and three 3-year-old male cattle-yaks (*n* = 3) in the same fattening base, Xiahe County, Gannan Tibetan Autonomous Prefecture (N 34°51′, E 102°26′). All animals were castrated through surgery. Before the operation, the reproductive organs were sterilized with iodophor (Sinopharm Chemical Reagent Co. Ltd., Shanghai, China) to prevent infection. Post-castration, the wound sites were sutured and penicillin ointment was applied twice a day for three days. Testes were subsequently cleaned using 75% ethanol (Sinopharm Chemical Reagent Co. Ltd., Shanghai, China), followed by phosphate-buffered saline (PBS) (Solarbio Science and Technology Co. Ltd., Beijing, China) with penicillin–streptomycin (Thermo Fisher Scientific, Waltham, MA, USA). The tunica albuginea was removed from each testis, and the two testes of each sample were cut into ~6-mm^3^-sized cubes using scissors. Some cubes were fixed in 4% paraformaldehyde (Solarbio Science and Technology Co. Ltd., Beijing, China), followed by paraffin embedding, and the remaining were snap-frozen in liquid nitrogen (−196 °C) for RNA and protein extraction.

### 2.3. Testes Measurement

The testes were wiped to remove any contaminants or substances after harvesting. Then, the testes were placed on white paper, which helped in obtaining a more accurate reading. After that, the length of the testes was measured from the top to the bottom using a ruler. We ensured that the ruler was placed straight and aligned with the length of the testes to obtain an accurate measurement (Table 1).

### 2.4. Hematoxylin and Eosin (H&E) Staining Assays

The fixed samples were stored overnight and then transferred into 70% ethanol for long-term storage. For paraffin embedding, the sections were dewaxed and rehydrated. Following this, they were stained with Hematoxylin and Eosin (H&E) solutions, dehydrated, and vitrified (Solarbio Science and Technology Co. Ltd., Beijing, China). Finally, the sections were sealed with neutral gum (Solarbio Science and Technology Co. Ltd., Beijing, China) and imaged using an E100 microscope (Nikon Corporation, Japan).

### 2.5. Reverse-Transcription Quantitative Polymerase Chain Reaction (RT-qPCR) Assays

Total RNA was extracted using a reagent kit from Tiangen Biotech (Beijing, China), following the instructions of the manufacturer. Complementary DNA synthesis was performed using the PrimeScript reverse transcription reagent kit (Takara, Japan). Primers (Table 2) were designed using the Primer3 Plus online software (https://www.primer3plus.com/, accessed on 16 March 2023) and synthesized by Sangon Biotech (Beijing, China). Reverse-transcription quantitative polymerase chain reaction (RT-qPCR) was performed using a QuantStudio 1 Plus system (Thermo Fisher Scientific, Waltham, MA, USA). Actin beta (ACTB) was used as the internal control, with yak testes as the control sample. The difference in gene expression was assessed by the 2^−ΔΔCt^ method.

### 2.6. Immunohistochemistry (IHC) Assays

The paraffin-embedded slices were dewaxed using the xylene solution (Sinopharm Chemical Reagent Co. Ltd., Shanghai, China) and rehydrated in absolute ethanol (Sinopharm Chemical Reagent Co. Ltd., Shanghai, China). After washing in distilled water, antigens were retrieved using a citric acid buffer (Servicebio Co. Ltd., Wuhan, China) in a microwave oven. Following this, the slices were washed with PBS, treated with 3% hydrogen peroxide (Annjet High-Tech Disinfection Technology Co. Ltd., Shandong, China) to block endogenous peroxidase activity, and washed again. To prevent non-specific binding, the testicular tissues were blocked with 3% bovine serum albumin (BSA) (Jing Ri Jin Dian Sci-tech Co. Ltd., Beijing, China). Primary antibodies were added to tissues and incubated at 4 °C overnight. After washing, the tissues were incubated with secondary antibodies. The following antibodies were used: rabbit anti-METTL14 (Bioss; 1:250); rabbit anti-ALKBH5 (Bioss; 1:250); rabbit anti-FTO (Bioss; 1:250); rabbit anti-YTHDF2 (Sloarbio; 1:250); and horseradish peroxidase-conjugated secondary antibody (goat anti-rabbit immunoglobulin G) (Servicebio; 1:200). The slices were stained using the diaminobenzidine solution (Servicebio Co. Ltd., Wuhan, China) and washed with water. Next, the nuclei were dyed with hematoxylin (Servicebio Co. Ltd., Wuhan, China). Finally, the sections were dehydrated, sealed, and imaged using an E100 microscope (Nikon Corporation, Japan).

### 2.7. Western Blotting Assays

The tissue samples were ground in liquid nitrogen, and total proteins were extracted using the radioimmunoprecipitation assay lysis buffer (Solarbio Science and Technology Co. Ltd., Beijing, China). Following this, the lysates were centrifuged at 12,000× *g* for 30 min. Protein concentrations were measured using the Bicinchoninic Acid Protein Assay Kit (Solarbio Science and Technology Co. Ltd., Beijing, China). The protein samples were mixed with 5× loading buffer (Solarbio Science and Technology Co. Ltd., Beijing, China) at a ratio of 4:1 and incubated in water (98 °C) for 10 min. Briefly, 30 μg of proteins from each sample was separated by sodium dodecyl sulfate–polyacrylamide gel electrophoresis (Bio-Rad, Hercules, CA, USA) and then transferred from the gel onto a polyvinylidene fluoride membrane (Millipore, MA, USA). Following this, the membrane was blocked with 5% (*m*/*v*) nonfat milk (Solarbio Science and Technology Co. Ltd., Beijing, China) diluted in 1× Tween/Tris-buffered saline (TBST) (Solarbio Science and Technology Co. Ltd., Beijing, China) at 25 °C for 2 h, and the membrane was washed for 10 min thrice. Next, the membrane was incubated with primary antibodies overnight at 4 °C, and after washing with TBST for 10 min thrice, the membrane was incubated with secondary antibodies for 2 h. The following antibodies were used: rabbit anti-METTL14 (Bioss; 1:2000), rabbit anti-ALKBH5 (Bioss; 1:2000), rabbit anti-FTO (Bioss; 1:2000), rabbit anti-YTHDF2 (Sloarbio; 1:2000), rabbit anti-ACTB (Bioss; 1:5000) antibodies, and horseradish peroxidase-conjugated secondary antibody (goat anti-rabbit immunoglobulin G) (Bioss; 1:20,000). After the incubation of the secondary antibody, the ECL Plus hypersensitive luminescent liquid (Solarbio Science and Technology Co. Ltd., Beijing, China) was added to the membrane. Finally, the membrane was imaged using ephoto (Genscript Biotech Corporation, Nanjing, China), and the protein levels were semi-quantified using the Image J 2.3.0 software.

### 2.8. Immunofluorescence Assays

The frozen slices were taken out from the ultra-low-temperature refrigerator and placed in a wet box until they returned to room temperature. After washing with PBS for 5 min, the tissues were blocked with PBS containing 5% BSA (Jing Ri Jin Dian Sci-tech Co. Ltd., Beijing, China) and 0.5% triton-100 (Solarbio Science and Technology Co. Ltd., Beijing, China). Next, the slices were placed at room temperature for 1 h. The following primary antibodies were used: rabbit anti-METTL14 (Bioss; 1:200), rabbit anti-ALKBH5 (Bioss; 1:200), rabbit anti-FTO (Bioss; 1:200), and rabbit anti-YTHDF2 (Proteintech; 1:200). After washing, the slices were incubated with primary antibodies that were diluted in a blocking buffer at 4 °C overnight. Following this, the residual primary antibodies were fully washed thrice with PBS for 15 min. The sections were then incubated with the secondary antibody (Cy3 conjugated goat anti-rabbit immunoglobulin G) (Servicebio; 1:300) at 25 °C for 2 h. Finally, the slices were washed for 10 min thrice. DAPI (4′,6-diamidino-2-phenylindole) (Beyotime Biotechnology Co. Ltd.; Shanghai, China) was added to the sections to stain the nucleus and then photographed under a fluorescence microscope (E100, Nikon Corporation, Japan). The excitation wavelength was 560 nm, emission wavelength was 590 nm.

### 2.9. Statistical Analyses

GraphPad Prism 8.3.0 was used for all statistical analyses. Data were compared via t-tests and were presented as mean ± standard deviation. The *p*-value of <0.05 was set as the threshold of significance.

## 3. Results

### 3.1. Morphological and Histological Differences in Testes

**Figure 1:**
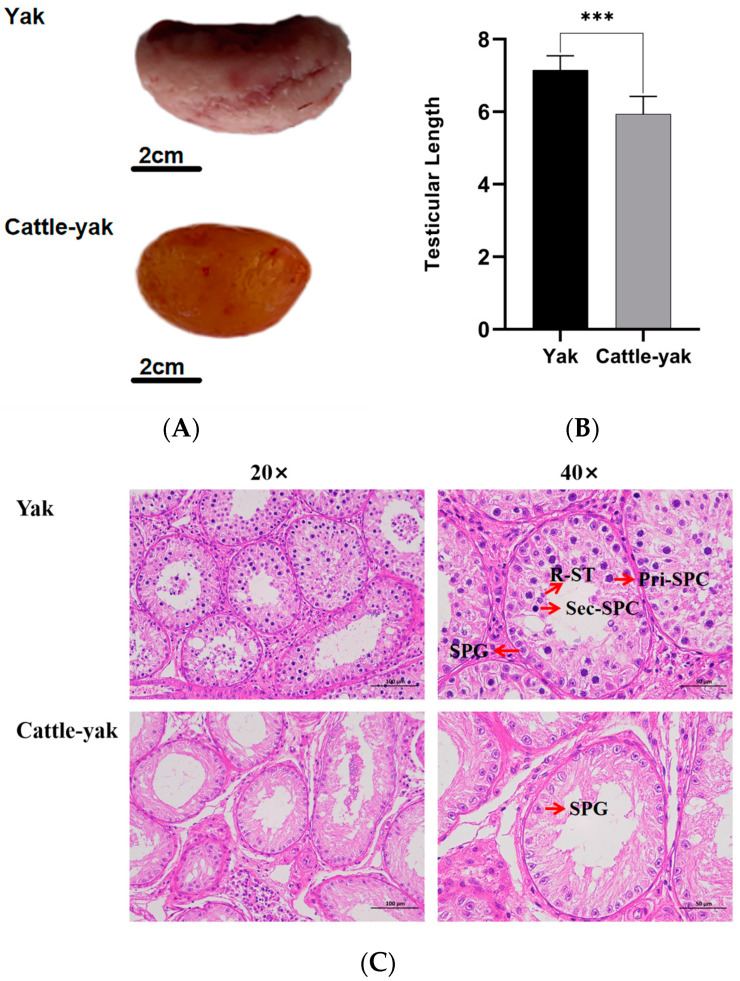
(**A**) Morphological, (**B**) testicular length, and (**C**) histological observations of testes of yak and cattle-yak. SPG, spermatogonia; Pri-SPC, primary spermatocyte; Sec-SPC, secondary spermatocyte; and R-ST, round spermatid. ***, *p* < 0.005.

Notably, the testes of sterile male cattle-yaks were smaller (Figure 1A), with the testicular length being markedly shorter than that of yaks (*p* < 0.005) (Figure 1B). Histological analysis revealed that the basal membrane of the cattle-yak testicular tissue was crumpled, with severe internal vacuolation (Figure 1C). Additionally, nearly all types of sperm cells were present in seminiferous tubules of the yak, including SPG (spermatogonia), Pri-SPC (primary spermatocyte), Sec-SPC (secondary spermatocyte), and R-ST (round spermatid), but only the SPG single cell layers were present in cattle-yak testes. This aberrant morphology of testes might be associated with male sterility in cattle-yaks.

### 3.2. Expression of m^6^A-Associated Genes in the Testicular Tissue

**Figure 2:**
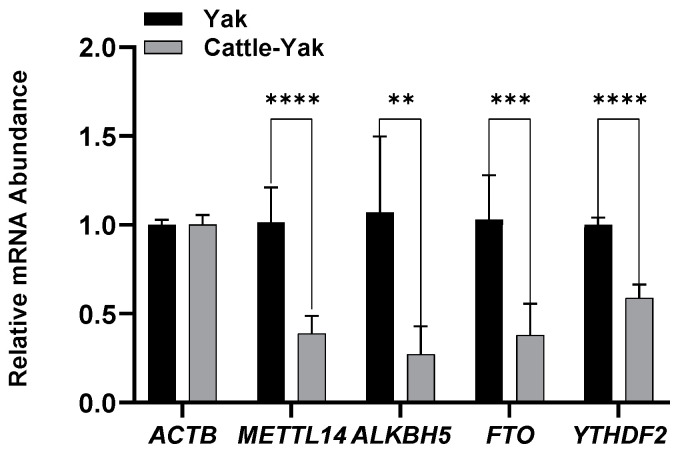
Relative mRNA expression of *ACTB*, *METTL14*, *ALKBH5*, *FTO*, and *YTHDF2*. Data are presented as mean ± standard deviation (*n* = 6; 3 yaks and 3 cattle-yaks; each sample represents 2 technical replicates); **, *p* < 0.01; ***, *p* < 0.005; and ****, *p* < 0.001. mRNA, messenger RNA; ACTB, actin beta; METTL14, methyltransferase-like 14; ALKBH5, alpha-ketoglutarate-dependent hydroxylase homolog 5; FTO, fat mass and obesity associated; and YTHDF2, YTH N^6^-methyladenosine RNA binding protein F2.

mRNA expressions were analyzed through RT-qPCR, and a considerable reduction in *METTL14* (*p* < 0.001), *ALKBH5* (*p* < 0.01), *FTO* (*p* < 0.005), and *YTHDF2* (*p* < 0.001) expression in cattle-yak testes was detected (Figure 2).

### 3.3. Protein Expression of m^6^A-Associated Genes

#### 3.3.1. Western Blotting Assay

**Figure 3:**
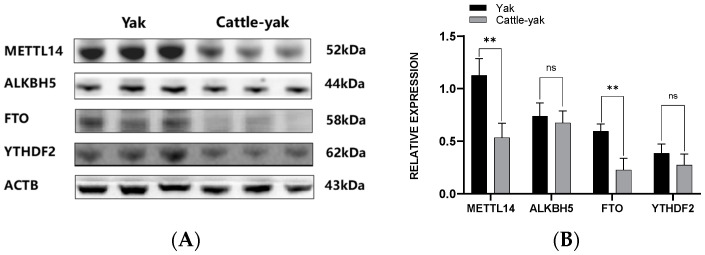
(**A**) Protein expression of METTL14, ALKBH5, FTO, and YTHDF2. (**B**) Relative expression of METTL14, ALKBH5, FTO, and YTHDF2. Data are presented as mean ± standard deviation; ns, no significance; **, *p* < 0.01. ACTB, actin beta; METTL14, methyltransferase-like 14; ALKBH5, alpha-ketoglutarate-dependent hydroxylase homolog 5; FTO, fat mass and obesity-associated; and YTHDF2, YTH N^6^-methyladenosine RNA binding protein F2.

The expression of the four proteins was semi-quantified using the Image J 2.3.0 software. Notably, METTL14 (*p* < 0.01) and FTO (*p* < 0.01) protein levels were markedly lower in cattle-yak testes. Furthermore, ALKBH5 and YTHDF2 levels showed a decreasing trend, without significance (Figure 3).

#### 3.3.2. Immunohistochemistry (IHC) Assay

**Figure 4:**
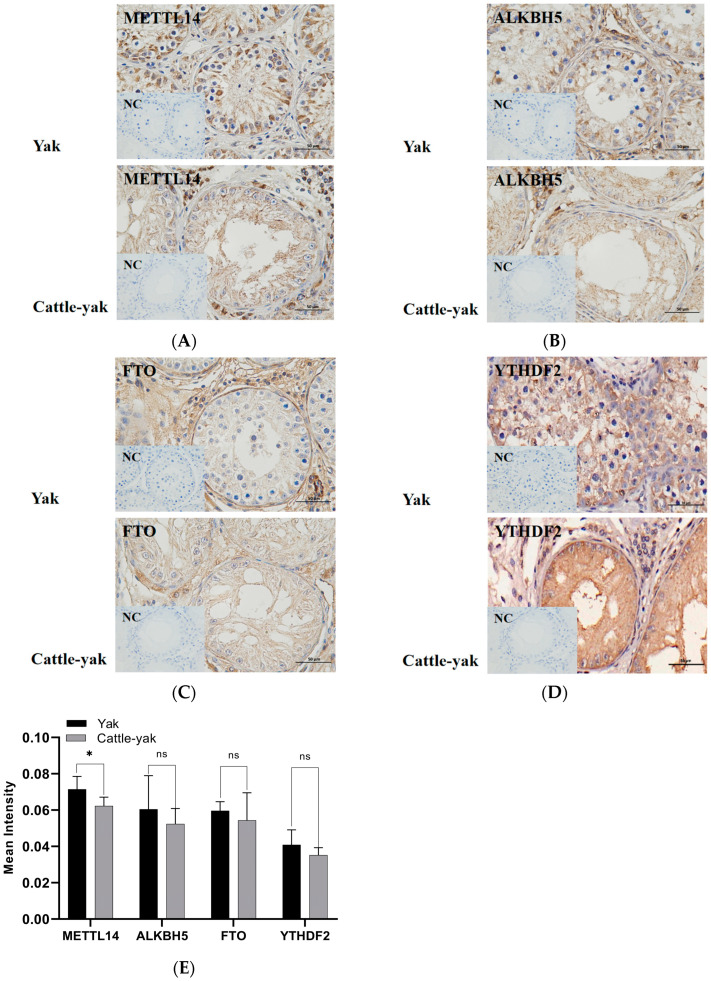
Immunohistochemical representative images of (**A**) METTL14, (**B**) ALKBH5, (**C**) FTO, and (**D**) YTHDF2 antibody-stained testes from the yak and cattle-yak. Insets show the NC in which primary antibodies were replaced with phosphate-buffered saline. NC, negative control; METTL14, methyltransferase-like 14; ALKBH5, alpha-ketoglutarate-dependent hydroxylase homolog 5; FTO, fat mass and obesity-associated; and YTHDF2, YTH N^6^-methyladenosine RNA binding protein F2. Scale bar, 50 μm (40×). (**E**) The expressions of METTL14, ALKBH5, FTO, and YTHDF2 were examined by immunohistochemical staining. Data are presented as mean ± standard deviation (*n* = 6; *n* = 6; 3 yaks and 3 cattle-yaks; each sample represents 2 technical replicates); ns, no significance; *, *p* < 0.05.

Immunohistochemistry (IHC) results showed that m^6^A-related genes were widely expressed in the testicular tissue. Gray-scale analysis of positive regions revealed that METTL14 (*p* < 0.05), ALKBH5, FTO, and YTHDF2 protein levels declined in the testes of cattle-yaks, along with the decrease of germ cells in seminiferous tubules (Figure 4).

#### 3.3.3. Immunofluorescence Assay

**Figure 5:**
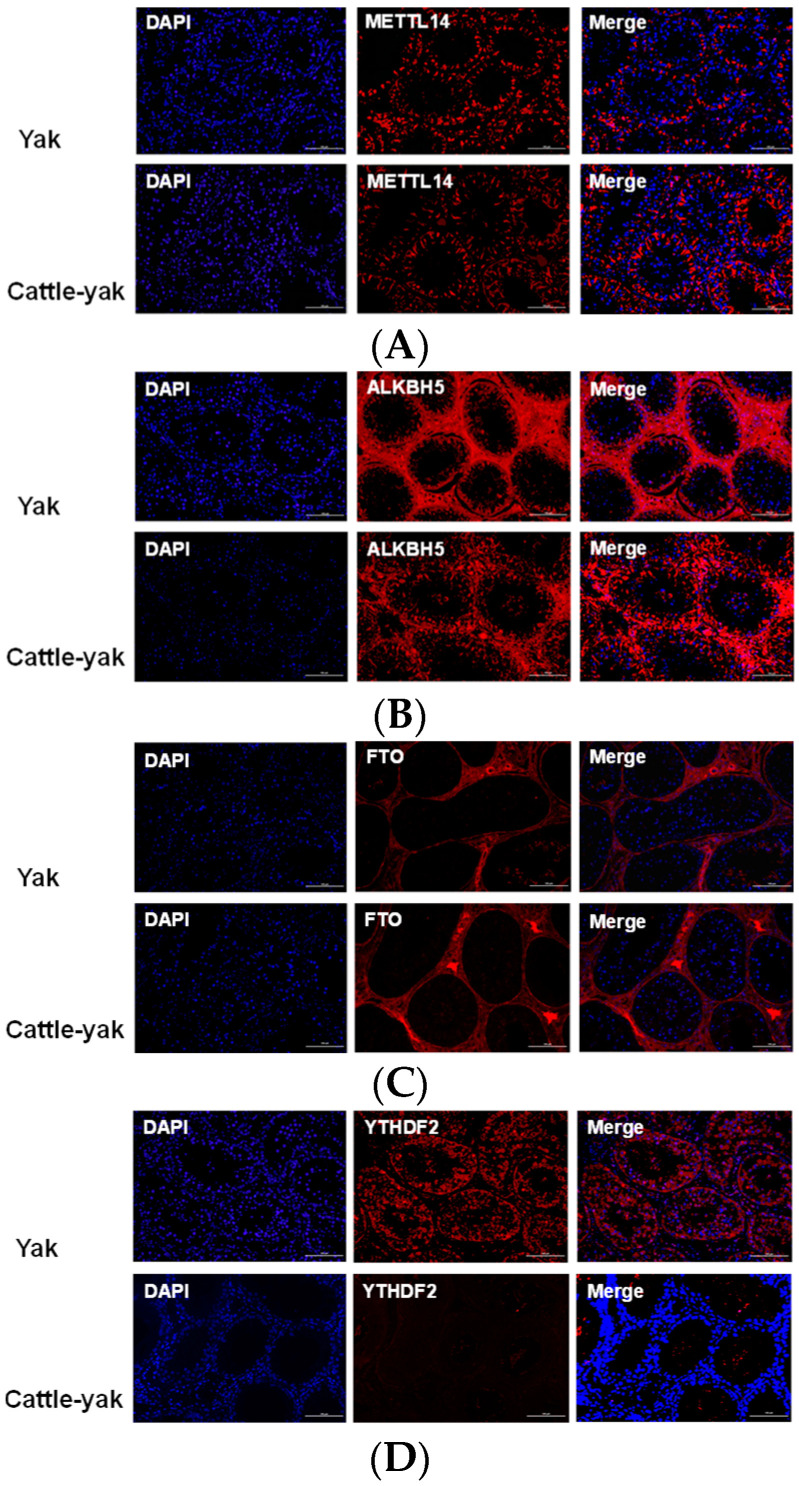
Immunofluorescence representative images of (**A**) METTL14, (**B**) ALKBH5, (**C**) FTO, and (**D**) YTHDF2 antibody-stained testes from the yak and cattle-yak. Scale bar, 100 μm (20×). DAPI, 4′,6-diamidino-2-phenylindole; METTL14, methyltransferase-like 14; ALKBH5, alpha-ketoglutarate-dependent hydroxylase homolog 5; FTO, fat mass and obesity associated; and YTHDF2, YTH N^6^-methyladenosine RNA binding protein F2.

Notably, immunofluorescence results showed that ALKBH5 was highly expressed in the testicular tissue. With the notable decrease in the number of germ cells in cattle-yak testes, the fluorescence signals also weakened accordingly (Figure 5).

## 4. Discussion

Cattle-yaks exhibit considerable heterosis compared with yaks, including a rapid growth period, robust adaptability, and high meat and milk yield. Nevertheless, owing to reproductive isolation, the heterosis cannot be stabilized through crossbreeding, which decreases the utilization value of cattle-yaks. This issue has garnered considerable attention from the scientific community. This study revealed that m^6^A-related genes were differentially expressed in sterile male cattle-yaks, suggesting a potential role in hybrid male sterility.

Histological analysis revealed distinct differences in testicular structure between yaks and cattle-yaks. Analysis of stained testicular tissue sections showed that yaks had nearly all types of spermatogenic cells (SGs) in their seminiferous tubules, whereas cattle-yaks only had a few. Previously, Wang et al. [27] compared the testes of 8-month-old and 2-year-old yaks, and the results revealed that the younger yaks had some meiotic cells, whereas the older yaks had SGs of all developmental stages. Similarly, Cai et al. [28] and Wu et al. [29] observed that 12-month-old yaks had all types of SGs, whereas cattle-yak testes mainly contained SPG. These studies suggest that yaks gradually reach sexual maturity, nevertheless, male cattle-yaks cannot produce mature sperm despite reaching adulthood. The findings of this study confirmed that only SPG was present in cattle-yak testes, which is consistent with the findings of previous studies and validates the accuracy of our sampling strategy.

H&E staining results showed an abnormal morphology of seminiferous tubules in cattle-yak testes. Androgens are crucial for promoting spermatogenesis, male reproductive organ growth, and developing male secondary sex characteristics. Reportedly, FSH cells in the distal anterior lobe of the pituitary gland in male cattle-yaks exhibited nuclear malformations, cytoplasmic intrusions into the nucleus, and the presence of small secretory particles. These abnormalities directly affect FSH secretion negatively, resulting in smaller testes and abnormal seminiferous tubules where spermatogenesis occurs [1].

Spermatogenesis is a complex biological process involving the highly ordered proliferation and differentiation of germ cells. It encompasses the proliferation and differentiation of spermatogenic stem cells to sperms, meiosis completion, and spermatid-to-spermatozoa transformation. Meiosis is a special division way to produce gametes during sexual reproduction which is essential for mammals to reproduce. Research originally proposed disrupted expression of spermatogenesis genes as a causative of sterility [30]. RNA sequencing showed that the widespread misexpression in cattle-yaks was associated with spermatogenesis-related genes [31]. It has been found that some genes related to spermatogenesis are abnormally expressed in cattle-yak testes, such as *DAZL*, *BOULE*, *SYCP3*, *SYCP2*, *FKBP6*, *DDX4*, *DDX25*, *SLX4*, *HSF2*, etc. [32,33,34,35,36,37,38,39,40,41]. Overall, the down-regulation of such genes hindered the meiosis process and affected spermatogenesis progress.

Further studies showed that the numbers of proliferative gonocytes and undifferentiated SPG in cattle-yaks were significantly higher than those in yaks (*p* < 0.01) [42]. Further in vitro proliferation and differentially expressed gene (DEG) analysis of specific types of cells revealed that undifferentiated SPG of cattle-yaks exhibited defects in viability and proliferation/differentiation potentials. Comparative single cell RNA sequencing (scRNA-seq) and in vitro proliferation analysis of testicular cells indicated that not only meiotic arrest contributed to MHS of cattle-yak. The spermatogenic arrest of cattle-yaks may originate from the differentiation stage of undifferentiated SPG and niche cells of cattle-yaks may provide an adverse microenvironment for spermatogenesis [43]. The most significant annotated differences between yaks and cattle-yaks LCs_MCs (Leydig cells and myoid cells) were associated with cell-to-cell communication. While LCs_MCs of yaks regulated spermatogenic cells at the SPG, SPC, and ST levels, no such relationships were found between LCs_MCs and germ cells in cattle-yaks. This may suggest that the somatic niche of male cattle-yaks testes is a microenvironment that is ultimately not favorable for spermatogenesis [44]. The m^6^A modification is a pivotal regulator in mammalian spermatogenesis. Recent studies have shown the effects of m^6^A modifications on gene expression and RNA stability, both of which are crucial for the proper development of germ cells [15,16,18,45,46,47,48]. This underscores the importance of m^6^A in reproductive health and its potential implications for addressing sterility issues from cattle-yaks to other mammalian species.

Previous research indicated that the m^6^A methylation level of cattle-yaks was significantly lower than that of cattle (*p* < 0.01), and it was also lower than that of yaks, with no significant difference [19]. This study showed a considerable decrease in the expression of *METTL14* (*p* < 0.001), *ALKBH5* (*p* < 0.01), *FTO* (*p* < 0.005), and *YTHDF2* (*p* < 0.001) in cattle-yak testes, compared with those in yaks. The observed decreased expressions of specific m^6^A-related genes in sterile cattle-yaks indicate a possible disruption in gene expression regulation, which could contribute to impaired spermatogenesis. One potential mechanism by which m^6^A modification could affect sterility is through the regulation of genes involved in testicular development and function. Disruptions in these pathways could lead to abnormal sperm production, as evidenced by the reduced testicular size and altered histological features observed in sterile males. A previous study demonstrated abnormal m^6^A methylation modification levels of a large number of genes, including apoptosis-related genes, autophagy-related genes, meiosis-related genes, etc. [49]. While this study provides valuable insights into the role of m^6^A modification in male cattle-yak sterility, the relatively small sample size and focus on specific genes limit the generalizability of the findings. The findings underscore the importance of m^6^A modification in hybrid male sterility, opening new avenues for research into the molecular basis of reproductive isolation in hybrids. Further studies should explore the full spectrum of m^6^A-modified genes and their regulatory networks in cattle-yaks.

## 5. Conclusions

Herein, the histological changes in the testes of both domestic yaks and cattle-yaks are shown. Furthermore, this study demonstrates that m^6^A-related genes are differentially expressed in sterile male cattle-yaks, suggesting a significant role of m^6^A modification in hybrid male sterility. By focusing on the role of m^6^A modification in male cattle-yak sterility, this study adds a novel dimension to our understanding of the molecular mechanisms underlying hybrid male sterility. Future research should expand on these findings by exploring a broader range of epigenetic modifications and their interactions with genetic factors in hybrid male sterility. Additionally, studies involving larger sample sizes and diverse hybrid populations would be valuable in confirming and extending these results. Ultimately, understanding the role of m^6^A modification in male cattle-yak sterility not only advances our knowledge of cattle-yak biology but also has the potential to inform breeding strategies that could overcome reproductive barriers in hybrids, enhancing their viability and productivity.

## Figures and Tables

**Table 1:** Morphological parameters of testes.

Testicular Length (cm)	Yak	Cattle-Yak
1	7.3	6.4
2	7.1	6
3	6.6	5.5
4	6.8	5.2
5	7.5	6.4
6	7.6	6.1
mean ± SD	7.150 ± 0.3937	5.933 ± 0.4885 ***

SD, standard deviation; ***, *p* < 0.005.

**Table 2:** Primer sequences used for reverse-transcription quantitative polymerase chain reaction.

Primer Name	Primer Sequences (5′–3′)
*METTL14*-F	GCAGAAGTTACGTCGACAGC
*METTL14*-R	AGGTATCATAGGAAGCCCTGC
*ALKBH5*-F	CGTGACTGTGCTCAGTGGATA
*ALKBH5*-R	CGGGGTGCATCTAATCTCGT
*FTO*-F	GAGCGCGAAGCTAAGAAAAGA
*FTO*-R	TCTGTGCATCAAGGATGGCT
*YTHDF2*-F	GGCAGTGGGTTCGGTCATAA
*YTHDF2*-R	ACCGAAGCTTCTCCAAGACG
*ACTB*-F	CACAGGCCTCTCGCCTTC
*ACTB*-R	ATCATCCATGGCGAACTGGT

*ACTB*, actin beta; *ALKBH5*, alpha-ketoglutarate-dependent hydroxylase homolog 5; *FTO*, fat mass and obesity-associated Protein; *METTL14*, methyltransferase-like 14; *YTHDF2*, YTH N6-methyladenosine RNA binding protein F2.

## Data Availability

The datasets produced or analyzed during the present study are available from the corresponding author upon reasonable request.
